# An Unusual Case of Meckel Diverticulitis Misdiagnosed as an Infected Urachal Cyst

**DOI:** 10.3390/medicina57050495

**Published:** 2021-05-13

**Authors:** Ioana Anca Stefanopol, Magdalena Miulescu, Liliana Baroiu, Aurelian-Dumitrache Anghele, Dumitru Marius Danila, Zina Tiron

**Affiliations:** 1 Department of Morphological and Functional Sciences, Faculty of Medicine and Pharmacy, “Dunărea de Jos” University, 800216 Galați, Romania; ancaflorea1969@yahoo.com; 2 Cardiorespiratory and Neuromotor Functional Exploration Laboratory, Faculty of Medicine and Pharmacy, “Dunărea de Jos” University, 800216 Galați, Romania; 3 Clinical Medical Department, Faculty of Medicine and Pharmacy, “Dunărea de Jos” University, 800216 Galați, Romania; liliana.baroiu@ugal.ro (L.B.); zina.tiron@ugal.ro (Z.T.); 4 Clinical Surgical Department, Faculty of Medicine and Pharmacy, “Dunărea de Jos” University, 800216 Galați, Romania; aurelian.anghele@ugal.ro (A.-D.A.); marius.danila@ugal.ro (D.M.D.)

**Keywords:** Meckel’s diverticulum, urachal cyst, children, embryology, management

## Abstract

*Introduction:* Meckel’s diverticulum (MD), a remnant of the omphaloenteric duct, is among the most frequent intestinal malformations. Another embryonic vestige is the urachus, which obliterates, becoming the median umbilical ligament; the failure of this process can lead to a urachal cyst formation. We present a case of Meckel diverticulitis misdiagnosed as an infected urachal cyst. *Presentation of case:* A 16-year-old girl presented with hypogastric pain, fever and vomiting. She had undergone an appendectomy 6 years prior and no digestive malformation had been documented. In the last 2 years, she had 3 events of urinary tract infections with Escherichia coli, and anabdominal ultrasound discovered a 28/21 mm hypoechogenic preperitoneal round tumor, anterosuperior to the bladder. We established the diagnosis of an infected urachal cyst, confirmed later by magnetic resonance imaging. Intraoperative, we found MD with necrotic diverticulitis attached to the bladder dome. *Discussions:* Meckel’s diverticulum and urachal cyst (UC) are embryonic remnants. Both conditions are usually asymptomatic, being incidentally discovered during imaging or surgery performed for other abdominal pathology. Imaging diagnosis is accurate for UC, but for MD they are low sensitivity and specificity. For UC treatment, there is a tendency to follow an algorithm related to age and symptoms, but there is no general consensus on whether to perform a routine resection of incidentally discovered MD. *Conclusion:* Preoperatory diagnosis of MD represents a challenge. We want to emphasize the necessity of a thorough inspection of the small bowel during all abdominal surgical interventions and MD surgical excision regardless of its macroscopic appearance. These two actions seem to be the best prophylaxis measures for MD complications and consequently to avoid emergency surgery, in which case more extensive surgical procedures on an unstable patient may be needed.

## 1. Introduction

Both UC and MD are embryologic vestiges originating in the yolk sac (endoderm). The umbilical cord contains various elements such as the allantois and the omphaloenteric duct. The allantois obliterates, forming the urachus, while the omphaloenteric duct resorbs completely [1,2]. If these processes fail, some anomalies may arise, including UC or MD [3]. While MD is one of the most frequent bowel malformations, with a 2% incidence, UC is exceedingly rare (1 in 300,000 births) [4]. Clinically, both conditions are usually asymptomatic, being discovered incidentally during imaging investigations or abdominal surgery; they become symptomatic when they complicate [4,5].

Regarding the imaging diagnosis methods, UC can be found on an abdominal ultrasound (US), but in MD cases, the correct diagnosis is provided by laparotomy or laparoscopy [3,4,5]. Because MD is relatively frequent, and its positive diagnosis is extremely difficult, a deliberate search for MD is advised during all abdominal surgical interventions. For incidentally discovered MD, prophylactic resection still remains controversial, but other studies advise it for all pediatric patients [6,7].

We report a challenging case of a 16-year-old girl, presenting with symptoms and imaging investigations matching with an infected UC, but during surgical intervention, we found Meckel diverticulitis attached to the bladder.

## 2. Case Presentation

A 16-year-old girl showed up at a general practitioner with a fever (39 °C), median subumbilical pain and dysuria. In the last 2 years, she had experienced 3 events of urinary tract infection (UTI) with Escherichia coli. Associated reno-urethral malformations were not documented during an US. The recommendation was to start treatment with ciprofloxacin and ibuprofen. Because her fever and pain persisted after 5 days, she was referred to our hospital. She complained of hypogastric pain with fever (39 °C), nausea and vomiting. She had been operated on 6 years prior for acute appendicitis (open surgery) and no digestive malformation was documented. The menarche was at the age of 14 years, but during the last 6months, she had experienced menstrual irregularity (vaginal bleeding every 2 weeks) and dysmenorrhea. Affirmatively she had no sexual activity. The physical examination revealed hypogastric tenderness without peritoneal signs, with median subumbilical warmth and erythema, but no drainage from the umbilicus was ever seen. Laboratory investigations found a high level of CRP (11.60 mg/dl), and urinalysisrevealedproteinuria (albumin: 140.02 mg/dl) and many epithelial cells, but the urine culture was sterile. The Addis–Hamburger test showed high levels of red and white blood cells and the rapid test for the detection of fecal occult blood in stool was positive.

**Figure 1:**
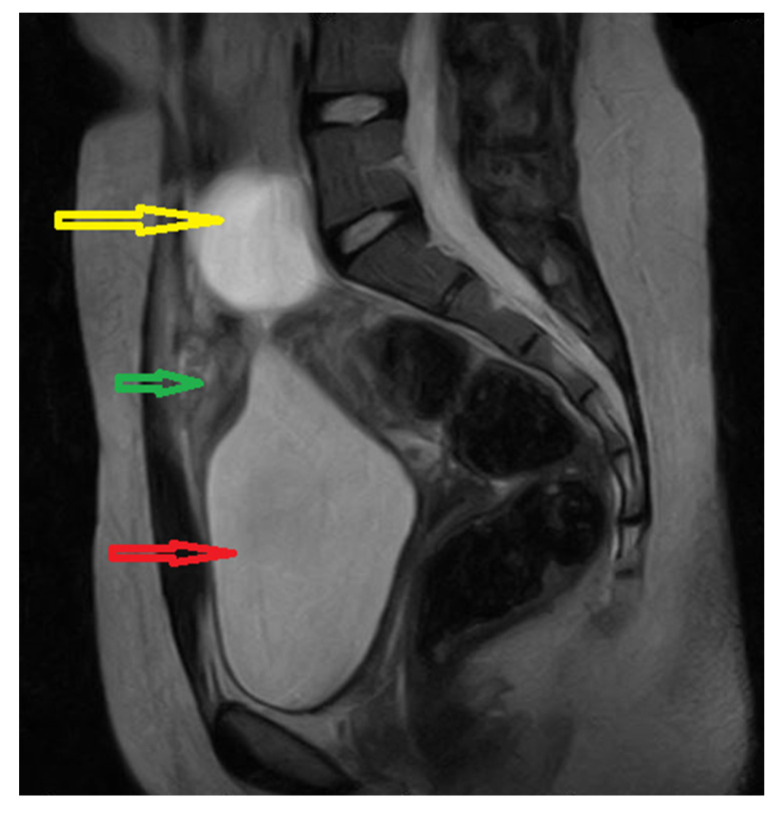
Pelvic MRI image: Sagittal T2-weighted yellow arrow: left ovarian cyst; green arrow: supposed urachal cyst; red arrow: urinary bladder.

The abdominal ultrasound (US) discovered a 28/21 mm hypoechogenic round tumor in the midline of the hypogastric region, anterosuperior to the bladder and posterior to the abdominal wall, with diffuse mural thickening. The uterus and both ovaries appeared to be normal. We decided to perform a pelvic MRI which showed a 16/18/32 mm inhomogeneous nodular lesion, apparently on the urachal tract; the bladder was in repletion, with mural thickening of the anterosuperior wall. The left ovary presented a 41/34 mm cyst, considered as a hemorrhagic corpus luteum (Figure 1).

**Figure 2:**
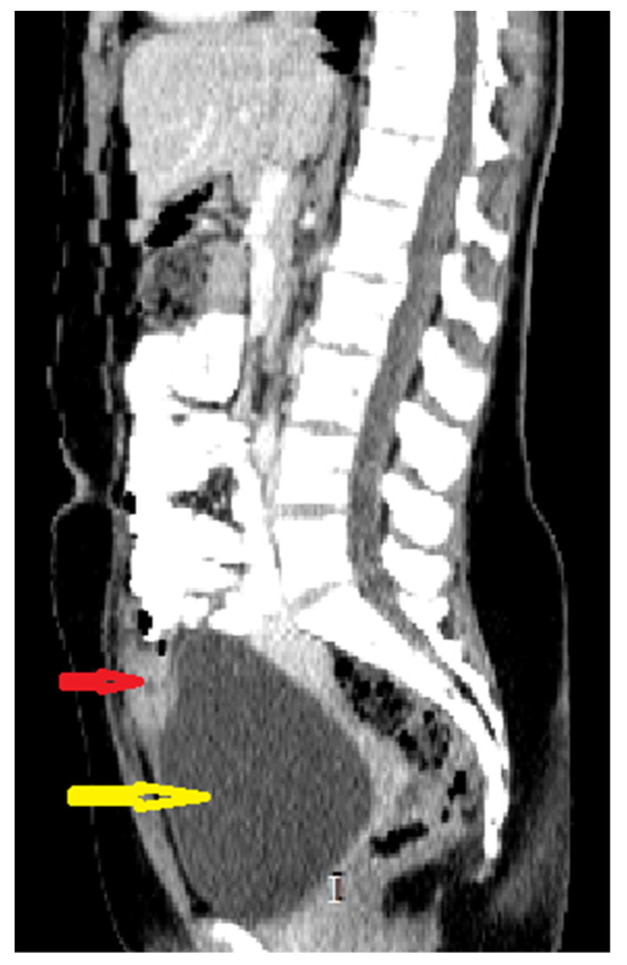
Sagittal CT scan reconstruction of abdomen and pelvis with intravenous and oral contrast: red arrow: supposed urachal cyst; yellow arrow: urinary bladder.

**Figure 3:**
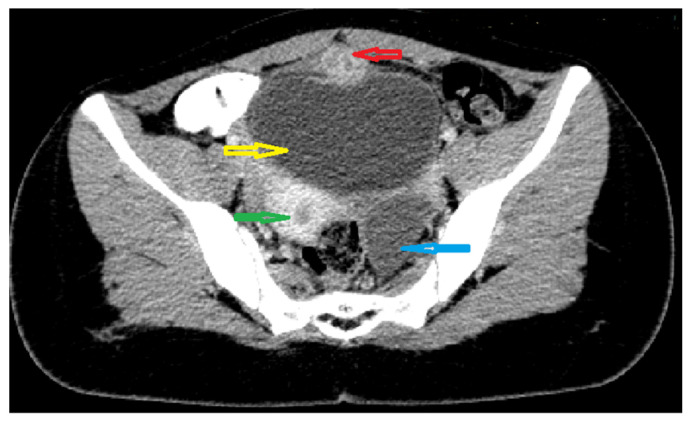
Axial CT scan of abdomen and pelvis with intravenous and oralcontrast: red arrow; supposed urachal cyst; yellow arrow: urinary bladder;green arrow: uterus; blue arrow: left ovarian cyst.

Because the radiologists requested additional investigations, an abdominal–pelvic CT scan with intravenous contrast was performed, adding an intense contrast capture in the periphery of the lesion with edema of the prevesical space (Figure 2 and Figure 3).

The diagnosis of an infected urachal cyst was established and we decided to manage the condition with a second generation of cephalosporins and observation. After 24 h, both symptoms and local inflammatory signs improved and 1 week later she was discharged from the hospital. After 10 days, the patient presented at the Emergency Department with signs of intestinal occlusion: infraumbilical pain, abdominal meteorism and vomiting. A new abdominal US was performed which showed the same cystic tumor as before, but between its anterosuperior pole and the prevesical fat there was a canal-like structure 28 mm long and 7 mm diameter, which was supposed to be the small intestine adherent to the infected UC.

We decided on an open-surgery intervention via a median infraumbilical laparotomy. The rectus abdominis muscles and properitoneal fat were edematous. In the abdominal cavity, we found a 6 cm long and 2.5 cm wide case of MD covered by the omentum and adherent to the midline of the anterior abdominal wall. Between the necrotic tip of the MD and the bladder, there was a little abscess (the antibiogram showed the presence of Staphylococcus epidermidis). Because the inflammatory process had damaged the corresponding detrusor muscle, we resected that area, and the bladder was closed using 3-0 vicryl. For the removal of MD, we performed a segmental resectionanastomosis. Both ovaries were normal but the uterus was large, with thick walls. We placed a urinary catheter which was removed after 3 days. Pathology revealed acute inflammatory tissue without malignant or ectopic mucosal cells. Postoperative, the recovery was uneventful and the patient was discharged after 8 days. Three months later she had no more digestive or urinary symptoms and experienced her first normal menstrual cycle.

## 3. Discussions

The allantois is a caudal diverticulum of the yolksac. Around the fifth gestational month, it will be completely obliterated, becoming the median umbilical ligament located in the prevesical space of Retzius [1,2]. If this process of involution does not occur it may arise structural anomalies such as: urachal fistula/patent urachus (completely open urachal lumina from the umbilicus to the bladder), urachal diverticulum (the urachus persists at the bladder dome), urachal cyst (a segment of urachus persists without any communication) and urachal sinus (the urachus persists at the umbilical side) [2,4]. UC is the most frequent type of anomaly (69%).

UC is ordinarily small and silent, but bacterial contamination or epithelial degeneration can enlarge the lumen and cause symptoms [8]. In this situation, the patient had abdominal signs mimicking an acute abdomen or a urinary tract infection: abdominal pain, fever, dysuria, periumbilical erythema and palpable subumbilical mass [4]. The most common UC complication is cyst infection; other complications are intracystic bleeding, stones, bowel fistula, intraperitoneal rupture, bowel obstruction, malignancy and urinary tract infections [8]. For an accurate diagnosis, US is usually enough, but an MRI or CT may also be useful [4,8].

Regarding the UC treatment, there is a tendency to follow an algorithm related to age and symptoms. Patients under 1 year of age, even symptomatic, are treated medically under US surveydue to frequent spontaneous resolution; surgery is reserved for recurrent infections. For older children or adults, the treatment of choice for symptomatic UC is surgical excision because of the high rate of malignant degeneration. So far, surgical intervention has been open excision, but lately, the laparoscopic approach is preferred [2,4,8].

The omphaloenteric duct joins the midgut lumen to the yolk sac. It gradually obliterates and later resorbs completely. If the process fails it can causemany anomalies such as MD, an umbilical cyst, omphalomesenteric fistula or umbilical sinus [3]. MD represents 97–98% of omphaloenteric duct anomalies, meaning the persistence of its enteric side as a blind recess with its own blood supply, located on the antimesenteric border of the distal ileum usually within 15–120 cm (an average distance of 60–100 cm) from the ileocecal valve [6]. Histologically, MD is a true diverticulum containing all layers of the small intestine. Histopathologically, 45–80% of MD specimens present ectopic mucosal tissue, mainly gastric mucosa (33%), but other mucosal types such as duodenal, pancreatic, hepatobiliary and colonic have also been reported in the literature [3]. This heterotopic tissue explains gastrointestinal bleeding which is the most common symptom in pediatric patients [6,9]. Only 4–6% of patients with MD become symptomatic, whenever a complication occurs such as gastrointestinal bleedings, diverticulitis, obstruction, invagination, perforation, hernia, strangulation or malignant degeneration. In the pediatric population, the complication rate is higher [9]. Almost 50% of complicated MD cases are encountered in children aged under 2 years [3,5] and the most frequent symptom is painless bleeding, followed by intestinal obstruction. Diverticulitis is characteristic of adults [10]. Clinical signs are various and with little specificity; this is the reason why symptomatic MD is misdiagnosed as other conditions that could cause an acute abdomen [5].

Complementary imaging studies are of little value; abdominal ultrasound, contrast-enhanced CT scan, angiography ormagnetic resonance imaginghave low sensitivity and specificity, often producing false-negative or false-positive results. The most useful investigation for MD detection in children is considered to be the technetium-99m pertechnetate scan, with a sensitivity of 80–90% and specificity of 95%, but only if the case of MD has ectopic gastric mucosa. In fact, exploratory laparotomy or laparoscopy are the investigations that can establish a correct diagnosis [3,5].

Regarding treatment, there is no general consensus on whether to perform a routine resection of incidentally discovered MD [6]. However, for children with MD, there is a tendency toperform a prophylactic removal even if it is a fortuitous discovery, while in adults it is indicated only if there are risk factors such as: length greater than 2 cm, age < 40 years, male gender and macroscopically evident heterotopic mucosa [5,7]. Wheneversurgical treatment is chosen, using intravenous propofol and ketamine for analgosedation in the induction of anesthesia provides a valuable measure of preemptive analgesia [11].

Three procedures have been described: segmental (‘‘T’’-shaped) resectionanastomosis, wedge resection and tangential stapling; for the last two options there remains a small risk of leaving residual heterotopic tissues at the MD base [5].

In our case, we excluded MD pathology because the patient had been operated on for acute appendicitis and we supposed that the mandatory intestinal exploration certified its absence. Moreover, the symptoms were specific for an infected UC (hypogastric pain and tumor, fever, repeated UTIs and infraumbilical erythema). The clinical diagnosis was sustained by imaging investigations and consequently, we decided to adopt a conservative attitude, treating the infection with antibiotics. Even a week later when the patient returned with clinical signs of intestinal occlusion and the abdominal US showed the cystic tumor attached to a canal-like structure supposed to be the small intestine, we thought that the infected UC was the cause of the symptoms because of its inflammatory adhesions to the bowel. We were extremely surprised when we found a MD abscess with an important detrusor muscle destruction.

We searched in the literature and we discovered only one pediatric case of Meckel’s diverticulitis presented with periumbilical cellulitis due to its attachment to the anterior abdominal wall [10].

## 4. Conclusions

Because of its various clinical presentations and despite high-performance imaging investigations, MD diagnosis is still a challenge. The purpose of this case report is to remind the importance and necessity of a thorough inspection of the last 150 cm of the ileum whenever an abdominal intervention is performed, as well as to recommend MD surgical excision regardless of its macroscopic appearance. These two actions seem to be the best prophylaxis measures for MD complications and to consequently avoid emergency surgery, in which case more extensive surgical procedures on an unstable patient may be needed.

## Data Availability

All of the current data is available on request from the authors.
